# Mitochondrial targeting of human NADH dehydrogenase (ubiquinone) flavoprotein 2 (NDUFV2) and its association with early-onset hypertrophic cardiomyopathy and encephalopathy

**DOI:** 10.1186/1423-0127-18-29

**Published:** 2011-05-06

**Authors:** Hsin-Yu Liu, Pin-Chao Liao, Kai-Tun Chuang, Mou-Chieh Kao

**Affiliations:** 1 Institute of Molecular Medicine & Department of Life Science, National Tsing Hua University, 101, Sec. 2, Kuang-Fu Rd., Hsinchu 30013, Taiwan, R.O.C

## Abstract

**Background:**

NADH dehydrogenase (ubiquinone) flavoprotein 2 (NDUFV2), containing one iron sulfur cluster ([2Fe-2S] binuclear cluster N1a), is one of the core nuclear-encoded subunits existing in human mitochondrial complex I. Defects in this subunit have been associated with Parkinson's disease, Alzheimer's disease, Bipolar disorder, and Schizophrenia. The aim of this study is to examine the mitochondrial targeting of NDUFV2 and dissect the pathogenetic mechanism of one human deletion mutation present in patients with early-onset hypertrophic cardiomyopathy and encephalopathy.

**Methods:**

A series of deletion and point-mutated constructs with the *c-myc *epitope tag were generated to identify the location and sequence features of mitochondrial targeting sequence for NDUFV2 in human cells using the confocal microscopy. In addition, various lengths of the NDUFV2 N-terminal and C-terminal fragments were fused with enhanced green fluorescent protein to investigate the minimal region required for correct mitochondrial import. Finally, a deletion construct that mimicked the IVS2+5_+8delGTAA mutation in *NDUFV2 *gene and would eventually produce a shortened NDUFV2 lacking 19-40 residues was generated to explore the connection between human gene mutation and disease.

**Results:**

We identified that the cleavage site of NDUFV2 was located around amino acid 32 of the precursor protein, and the first 22 residues of NDUFV2 were enough to function as an efficient mitochondrial targeting sequence to carry the passenger protein into mitochondria. A site-directed mutagenesis study showed that none of the single-point mutations derived from basic, hydroxylated and hydrophobic residues in the NDUFV2 presequence had a significant effect on mitochondrial targeting, while increasing number of mutations in basic and hydrophobic residues gradually decreased the mitochondrial import efficacy of the protein. The deletion mutant mimicking the human early-onset hypertrophic cardiomyopathy and encephalopathy lacked 19-40 residues in NDUFV2 and exhibited a significant reduction in its mitochondrial targeting ability.

**Conclusions:**

The mitochondrial targeting sequence of NDUFV2 is located at the N-terminus of the precursor protein. Maintaining a net positive charge and an amphiphilic structure with the overall balance and distribution of basic and hydrophobic amino acids in the N-terminus of NDUFV2 is important for mitochondrial targeting. The results of human disease cell model established that the impairment of mitochondrial localization of NDUFV2 as a mechanistic basis for early-onset hypertrophic cardiomyopathy and encephalopathy.

## Background

Mammalian NADH:ubiquinone oxidoreductase (complex I) (EC 1.6.5.3) is the first, largest and most complicated respiratory complex in mitochondria [1]. It is one of the electrons entry sites in the oxidative phosphorylation system (OXPHOS), and catalyzes NADH oxidation, followed by transferring two electrons to ubiquinone [2]. To date, 45 different subunits have been identified in bovine heart mitochondrial complex I [3,4]. Among them, seven subunits of complex I, including ND1-6 and ND4L, are encoded by mitochondrial DNA, and the others are encoded by nuclear DNA [5]. In contrast, bacterial complex I (also called NDH-1) is much simpler. It contains only 13-14 unlike subunits [6]. These subunits of bacterial origins are conserved in mitochondrial complex I and considered as the "minimal" structure required for correct function. The two recently published crystal structures of the complete complex I from prokaryote *Thermus thermophilus *and eukaryote *Yarrowia lipolytica *indicated that this enzyme complex is L-shaped and separated into two arms: a hydrophobic arm embedded in the periplasm/the inner membrane and a hydrophilic arm protruding into the cytoplasm/the matrix [7,8]. The bacterial complex I possesses nine Fe-S clusters, including two [2Fe-2S] clusters (N1a and N1b) and seven [4Fe-4S] clusters (N3, N4, N5, N6a, N6b, N7 and N2), to manage the passage of two electrons [9]. According to the *T. thermophilus *model, the main pathway for electron transfer in complex I is NADH- FMN- N3- N1b- N4- N5- N6a- N6b- N2- quinine [10,11].

Human NADH dehydrogenase (ubiquinone) flavoprotein 2 (NDUFV2) subunit, also called 24-kDa, is one of the complex I core subunits which are very conserved from bacteria to mammals [12]. The *NDUFV2 *gene has been cloned and assigned to human chromosome 18p11.31-p11.2 [13]. The entire gene spans approximately 20 kb and contains 8 exons, and the expressed protein is homologous to 24-kDa of *Bostaurus *and *Neurospora crassa *[14], NuoE of *Escherichia coli *[15] and *Rhodobacter capsulatus *[16], NQO2 of *Paracoccus denitrificans *[17] and *T. thermophilus *[18], and NUHM of *Y. lipolytica *[19]. Human NDUFV2 contains a binuclear [2Fe-2S] cluster called N1a. This iron-sulfur cluster has a binding motif, Cys-(X)_4_-Cys-(X)_35_-Cys-(X)_3_-Cys, which is very conserved among orthologues [20]. Based on the crystal structure of the hydrophilic domain from *T. thermophilus *complex I, cluster N1a can accept electrons from FMN, but is unable to pass them to cluster N3, which is too far away from N1a [10,11]. One hypothesis suggests that cluster N1a may act as an antioxidant to accept the excessive electrons to prevent the generation of reactive oxygen species (ROS) [10,11].

The fungus *N. crassa *is an eukaryotic organism which is frequently used as a model to study the structure and function of complex I [21]. In the *N. crassa *studies, it was found that the lacking of 24-kDa subunit would reduce the levels of 51-kDa subunit (a homologous of human NDUFV1) and affect the NADH:ferricyanide reductase activity, suggesting that the 24-kDa subunit is essential for a proper assembly of 51 kDa subunit and complex I activity [14]. This phenotype may explain why the deficiency of NDUFV2 subunit has been associated with some neurodegenerative diseases, including Parkinson disease [22], Alzheimer's disease [23], Bipolar disorder, and Schizophrenia [24,25].

Most nuclear DNA-encoded mitochondrial proteins, including NDUFV2, are synthesized in the cytosol on free ribosomes as a precursor protein which carries a mitochondrial targeting sequence (MTS) for correct import. These mitochondrial preproteins are then transported into or across mitochondrial membranes with the help of several distinct complexes, including the translocase of outer membrane (TOM) complex and the translocase of inner membrane (TIM) complex [26,27]. The final location of the protein will be determined by the combined actions of the involved translocation pathway and the targeting message encoded within the protein. For most proteins targeted to the mitochondrial matrix and some of those destined for the intermembrane space and the inner membrane, a cleavable extension is frequently present in the N-terminus of the precursor protein. This sequence contains about 10-80 amino acid residues that have a high content of basic, hydrophobic and hydroxylated amino acids but a lack of negatively charged amino acids [28]. The positive residues are considered to play an important role in mitochondrial targeting, and are thought to assist the MTS across the inner membrane driving by the membrane potential. Having the potential to form amphiphilic α-helices is another common feature that is proposed for receptor recognition. The molecular structure of a general import receptor TOM20 interacting with a mitochondrial presequence suggests the importance of the amphiphilic α-helical structure and the involvement of hydrophobic residues in binding to this mitochondrial import receptor [29]. However, the result from an import study based on several artificial presequences fused with a passenger protein suggested that amphiphilicity is necessary for mitochondrial import but forming a helical structure may not be essential [30]. Except these characteristics, there is no sequence identity shared between MTSs, even between closely related orthologs. Most of the N-terminal MTSs are cleaved from precursors by the mitochondrial processing peptidase (MPP) in one step, some others are processed sequentially by MPP and the mitochondrial intermediate peptidase (MIP) in a two-step reaction [28]. Infrequently, the MTS can be found to be present at the C-terminus.

The mature 24-kDa of complex I has been purified from bovine heart and the primary structure of this protein has been partially determined [31]. According to the complementary DNA (cDNA) sequence of *NDUFV2 *and its close relatedness with the bovine sequence, the possible human precursor and mature sequences of NDUFV2 subunit were predicted [32]. In a recent report, a 4-bp deletion in intron 2 (IVS2+5_+8delGTAA) in the *NDUFV2 *gene has been shown to associate with patients with early-onset hypertrophic cardiomyopathy and encephalopathy [33]. This mutation altered the splicing donor site and caused the exon 2 missing in the mRNA of *NDUFV2*. The truncated RNA transcript is predicted to encode a shorter protein not only lacking part of the MTS but also losing the cleavage-processing site. Biochemical analyses indicated that patients with this mutation had a 70% reduction in the amount of NDUFV2 protein and a significant complex I deficiency [33]. Scientists have tried to simulate this exon 2 skipping mutation by deleting the corresponding region of orthologous *NUHM *gene in the obligate aerobic yeast *Y. lipolytica *[19]. Surprisingly, the results showed that this mutant was indistinguishable from normal cells in activity, inhibitor sensitivity and EPR signals of complex I in this yeast model.

The mitochondrial targeting of NDUFV2 has not been experimentally established. In the present study, a series of N-terminal truncated, C-terminal truncated and point-mutated constructs with the *c-myc *epitope tag were generated to identify the location and sequence features of MTS for NDUFV2 in human cells. In addition, various lengths of the NDUFV2 N-terminus and C-terminus were fused with enhanced green fluorescent protein (EGFP) to investigate the minimal functional region required for correct mitochondrial import. Finally, a deletion construct that mimics the IVS2+5_+8delGTAA mutation and would produce a shortened precursor protein lacking 19-40 residues in NDUFV2 was generated to dissect the pathogenetic mechanism of this mutation.

## Methods

### Cell and bacterial culture

T-REx-293 cells (Invitrogen, Carlsbad, CA, USA), human embryonic kidney cells with the tetracycline-regulated expression system, were cultured at 37°C and 5% CO_2 _with saturating humidity in Dulbeccos modified Eagle media (DMEM) which contained 10% fetal bovine serum (FBS), 100 U/ml penicillin and 100 μg/ml streptomycin. *Escherichia coli *DH5α strain and Top10F' strain were used for gene cloning, and the bacteria were grown in Luria Bertani (LB) media or on LB agar plates containing ampicillin (100 μg/ml) at 37°C.

### Plasmid construction

#### Construction of plasmids expressing full-length NDUFV2 proteins

The Mammalian Gene Collection (MGC) cDNA clone encoding human NDUFV2 (accession numbers NM_021074, clone number: MGC-15943, IMAGE: 3537815) was obtained from the I.M.A.G.E Consortium. The derived plasmid was used as the template for amplification by polymerase chain reaction (PCR) using *Pfu *DNA polymerase. The sequences of primers used are shown in Additional file 1-(1). The resultant fragment was then cloned into the pGEM-T vector (Promega, Madison, WI, USA) and the sequence was confirmed by sequencing. The resulting plasmid was digested with EcoRI/XhoI, and the DNA fragment containing the desired cDNA was then purified and ligated with the pcDNA4/TO/*myc*-His A vector (Invitrogen) using the same restriction sites to generate the pcDNA4-NDUFV2 expressing vector.

#### Construction of plasmids expressing truncated NDUFV2 proteins

The pcDNA4-NDUFV2 vector was used as the template for generation of its N-terminal deletion constructs (pcDNA4-△1-18 NDUFV2, pcDNA4-△1-32 NDUFV2 and pcDNA4-△1-50 NDUFV2) and C-terminal deletion constructs (pcDNA4-△183-249 NDUFV2 and pcDNA4-△198-249 NDUFV2). The sequences of primers used are shown in Additional file 1-(2). In addition, the construct (named pcDNA4-△19-40 NDUFV2) which mimics the human pathogenic IVS2+5_+8delGTAA mutation in *NDUFV2 *gene in patients with hypertrophic cardiomyopathy and encephalomyopathy was generated with the primers shown in Additional file 1-(3).

#### Construction of plasmids expressing various lengths of NDUFV2-EGFP

Using the pcDNA4-NDUFV2 plasmid as the template, various DNA fragments encoding different N-terminal proteins of NDUFV2 were designed and generated to fuse with *EGFP *gene in the pEGFP-N3 expression vector (Clontech Laboratories, Mountain view, CA, USA). The restriction enzyme sites used for this purpose were *Xho*I and *Eco*RI. These resulting constructs included NDUFV2 full-length (pEGFP-N3 NDUFV2_1-249_), pEGFP-N3 NDUFV2_1-32_, pEGFP-N3 NDUFV2_1-22_, pEGFP-N3 NDUFV2_1-21_, pEGFP-N3 NDUFV2_1-20 _and pEGFP-N3 NDUFV2_1-18_. The sequences of primers used are shown in Additional file 1-(4).

#### Construction of plasmids expressing NDUFV2 missense mutants

The pcDNA4-NDUFV2 was used as the template for introduction of missense mutations on basic, hydroxylated and hydrophobic residues in the first 1-32 amino acids of NDUFV2 using the site-directed mutagenesis methodology based on the QuickChange manual (Stratagene, La Jolla, CA, USA). All of the used primers are shown in Additional file 1-(5, 6, 7).

### Transient transfection and immunofluorescent staining

T-REx-293 cells were seeded in 24-well plates containing cover glasses. When cell growth reached approximately 60-70% confluency, TransIT-LT1 transfection Reagent (Mirus, Madison, WI, USA) pre-mixing with the desired plasmid was introduced for transfection. After 24-h incubation, the culture medium was removed and the fresh medium containing tetracycline to a final concentration of 0.5 μg/ml was added to the cell culture. Following 24 h of tetracycline induction at 37°C, cells were incubated with the growth medium containing 100 nM Mito Tracker Red (CMX-Ros; Molecular probe, Eugene, USA) for 30 min, followed by washing once in the phosphate-buffered saline (PBS) buffer. Next, cells were permeated and fixed with the acetone and methanol mixture (acetone: methanol = 3: 1 in volume proportion) for 5 min on ice. After fixation, cells were first incubated with growth media at room temperature for 2 h and then with diluted monoclonal mouse anti-c-*myc *antibody (Calbiochem, 1:100 dilution) at room temperature for 1 h. After 5 times of washing with the PBS buffer, the cells were incubated with goat anti-mouse IgG-FITC (Invitrogen, 1:100 dilution) at room temperature for another 1 h, and washed again by the PBS buffer. Finally, the cover glass was mounted with the VECTASHIELD Mounting Medium (Vector Laboratories, Burlingame, CA, USA). When the EGFP fusion constructs were applied for analyses, cells were fixed with 4 % paraformaldehyde in PBS for 15 min at room temperature and then permeabilized with 0.5 ml methanol for 5 min on ice. The following steps were executed as the procedure described for staining with antibodies. Immunofluorescence was visualized by the LSM510 laser scanning confocal microscope (Carl Zeiss, Oberkochen, Germany) using excitation and emission filters at 488 and 510 nm, respectively, for the FITC or EGFP signal, and 543 and 565 nm, respectively, for the Mito Tracker Red signal. The resulting images were merged for evaluation of co-localization. For assessing the efficiency of mitochondrial targeting, fusion protein (EGFP-fused or c-*myc*-tagged) import into mitochondria was monitored by confocal microscopy in at least 50 fusion protein-expressing cells, and quantified as the ratio of the number of cells in which the fusion protein was co-localized with mitochondria (labelled with Mito Tracker Red) relative to the total number of fusion protein-expressing cells. For each construct, the confocal image analysis was performed in three separate transfection experiments.

### Western blotting analyses and antibodies

For Western blotting analyses, T-REx-293 cells were transfected with the desired plasmids as described above. Cells with tetracycline induction were collected by trypsinization and centrifuged with 1000 × *g *force for 5 min at 4°C. The pellet was washed once and centrifuged at the same conditions for another 5 min. The collected pellet was then suspended with the lysis buffer (0.15 M NaCl, 5 mM EDTA pH 8, 1% Triton-X 100, 10 mM Tris-Cl, pH 7.4) for 20 min on ice and centrifuged at 12000 × *g *for 10 min at 4°C. The supernatant was transferred to a new eppendorf tube and the protein concentration was determined by the BCA protein assay kit (Thermo Scientific, Rockford, IL, USA). The 4× protein loading dye was then added to the supernatant and the resulting mixture was boiled for 5 min. Next, the proteins were separated by 10 or 15% SDS-PAGE (sodium dodecyl sulphate polyacrylamide gel electrophoresis) and transferred onto a polyvinylidene fluoride (PVDF) membrane at 350 mA constant current for 90 min. The membrane was then blocking with 5% skin milk in the PBS buffer at room temperature for 90 min and incubated with the diluted primary antibody at room temperature for 1 h. After three times of PBS washing for 10 min each, the membrane was then incubated with a proper secondary antibody at room temperature for 1 h, and followed by several washes using the PBS buffer. Finally, the enhanced chemiluminescence (ECL) system (PerkinElmer, Walcham, MA, USA) was applied for detection. The primary antibody used in this study included monoclonal mouse anti-c-*myc *antibody (Calbiochem, San Diego, CA, USA), monoclonal mouse anti-β-tubulin antibody (Santa Cruz Biotechnology, Santa Cruz, CA, USA), monoclonal mouse anti-β-actin antibody, (Novus Biologicals, Littleton, CO, USA) and monoclonal mouse anti-ATP synthase subunit α antibody (Invitrogen). The secondary antibody included goat anti-mouse IgG-HRP (Invitrogen).

### Subcellular fractionation

Subcellular fractionation of cells to separate mitochondrial and cytosolic fractions was conducted according to a published differential centrifugation method with some modifications [34]. T-REx-293 cells collected from three 10-cm culture dishes with trypsination were washed once with the PBS buffer and then resuspended in 1 ml hypotonic buffer (10 mM HEPES, 1 mM KH_2_PO_4_, 10 mM NaCl, 5 mM NaHCO_3_, 1 mM CaCl_2_, 0.5 mM MgCl_2 _and 5 mM EDTA). After incubation on ice for 5 min to promote hypotonic swelling, cells were homogenized by 30 up-and-down strokes with a glass homogenizer, followed by the addition of 100 μl 2.5 M sucrose to prevent organelles of cells from bursting. The homogenate was centrifuged at 1000 × *g *for 10 minutes at 4°C and the collected supernatant was transferred to a clean chilled tube for further centrifugation at 12000 × *g *for 15 min at 4°C. The supernatant representing the cytosolic fraction was collected without any treatment and stored at -20°C for later analyses. The pellet was washed once with the mitochondrial isolation buffer (250 mM sucrose, 0.1 mM EGTA and 20 mM HEPES, pH 7.4). The resulting pellet representing the mitochondrial fraction was finally resuspended in 40 μl PBS containing 0.1% SDS.

## Results

### The MTS of NDUFV2 was located at the N-terminus of the protein

**Figure 1:**
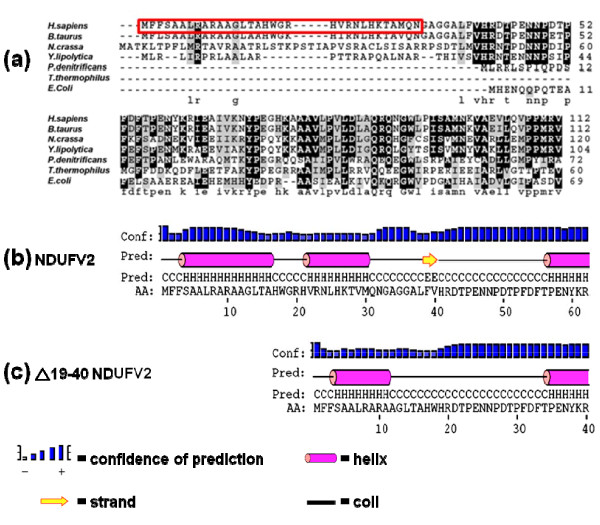
**Sequence comparison and secondary structure analysis of the N-terminal region of NDUFV2**. (a) Multiple sequence alignment of NDUFV2 proteins from different species. Sequence alignment was generated by EMBL-EBI Clustal W2 [47] and displayed by BOXSHADE server [48]. The abbreviations used are: *H. sapiens*, *Homo sapiens *NDUFV2 (UniProt: P19404); *B. Taurus*, *Bos taurus *24 kDa (UniProt: P04394); *N. crassa*, *Neurospora crassa *NUO-24 (UniProt: P40915); *Y. lipolytica*, *Yarrowia lipolytica *NUHM (UniProt: Q9UUT9); *P. denitrificans*, *Paracoccus denitrificans *NQO2 (UniProt: P29914); *T. thermophilus*, *Thermus thermophilus *Nqo2 (UniProt: Q56221); *E. coli*, *Escherichia coli *strain K12 NuoE (UniProt: P0AFD1). Residues identical to the consensus are highlighted in reversed-out lettering on a black background; residues not identical but similar to the consensus are shown on a grey-shaded background. (b) The secondary structure prediction of wild-type and NDUFV2 IVS2+5_+8delGTAA disease mutant. Secondary structure of the N-terminal region of NDUFV2 was predicted by the PSIPRED server [38]. H, α-helix; C, coil; E, strand.

**Figure 2:**
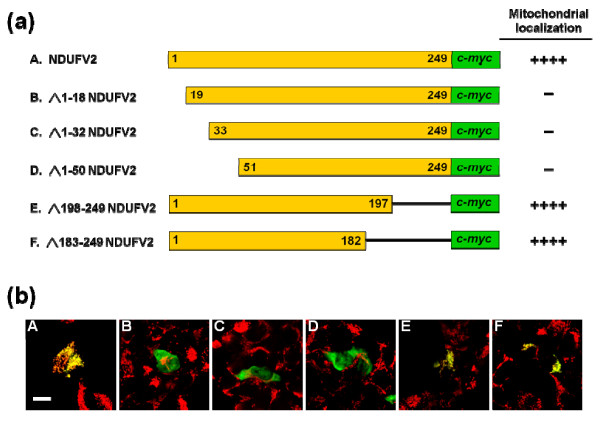
**Effects of NDUFV2 N-terminal and C-terminal truncation on mitochondrial targeting of the protein**. (a) The constructs generated to express full-length and truncated NDUFV2 proteins. Full-length NDUFV2 (A), N-terminal truncation (B, △1-18 NDUFV2; C, △1-32 NDUFV2; D, △1-50 NDUFV2) and C-terminal truncation (E, △198-249 NDUFV2; F, △183-249 NDUFV2) were fused with *c-myc *epitope tag, and expressed in T-REx-293 cells. The number of (+) symbols indicates that the proportion of cells exhibiting FITC fluorescence have a typical punctuated staining pattern and mitochondrial colocalization in (b). The (++++) symbol indicates all of the FITC fluorescence signals in transfected cells are fully colocalized with mitochondria. The (-) symbol indicates that there is no cell producing FITC fluorescence within the mitochondrial compartment. (b) The distribution of c-*myc *fusion proteins was detected by anti-c-*myc*-FITC antibody (green color) and mitochondria were labeled by Mito Tracker Red (red color). Only merged images are shown (colocalization of expressed protein and mitochondria is indicated by yellow signals). Photos A-F are corresponding to constructs A-F shown in (a). Scale bars = 10 * μ*m.

NDUFV2 is a nuclear-encoded mitochondrial protein which is assembled into the L-shaped complex I and is localized in the hydrophilic arm protruding into the matrix. Therefore, this protein is expected to be imported into mitochondria through a pathway specific for mitochondrial matrix proteins. Analyses of this protein by MitoProt II [35] suggested a 99.6% probability of mitochondrial targeting of NDUFV2. A very similar result was also obtained from the prediction from the TargetP server [36]. Protein sequence alignment of NDUFV2 from various species revealed that the proteins from eukaryotic species have a non-conserved region located at the N-terminus (Figure 1a). It has been predicted that the first 32 amino acids of NDUFV2 may be the MTS of this protein [32]. To test this prediction, full-length, various N-terminal and C-terminal deletion constructs were generated to determine the location and orientation of MTS in NDUFV2 (Figure 2a). A *c-myc *epitope tag was appended to the C-terminus of these constructs to facilitate detection and analysis by the immunofluorescent staining method. All of the designed constructs were successfully engineered from the NDUFV2 cDNA and confirmed by direct sequencing. After transient transfection, mouse anti-*c-myc *antibody was applied to detect the expressed proteins, and the Mito Tracker Red dye was used to mark the mitochondria in T-REx-293 cells. The results showed that the full-length NDUFV2 construct had a punctuated cytosolic staining pattern that was typically observed when mitochondria were immunostained, indicating applicable of the experimental strategy. When the C-terminal deletion constructs (pcDNA4-△183-249 NDUFV2 and pcDNA4-△198-249 NDUFV2) were individually transfected into T-REx-293 cells, both of the truncated proteins were still colocalized with mitochondria. However, N-terminal truncations of NDUFV2 including △1-18 NDUFV2, △1-32 NDUFV2 and △1-50 NDUFV2, all lost their mitochondrial localization (Figure 2b). These observations agree well with the suggestion from protein sequence alignment and the protein domain prediction programs, and indicate that the MTS of NDUFV2 is located at the N-terminus of the precursor protein.

### NDUFV2 was processed *in vivo *by proteolytic removal of the N-terminal MTS at a cleavage site around amino acid residue 32

**Figure 3:**
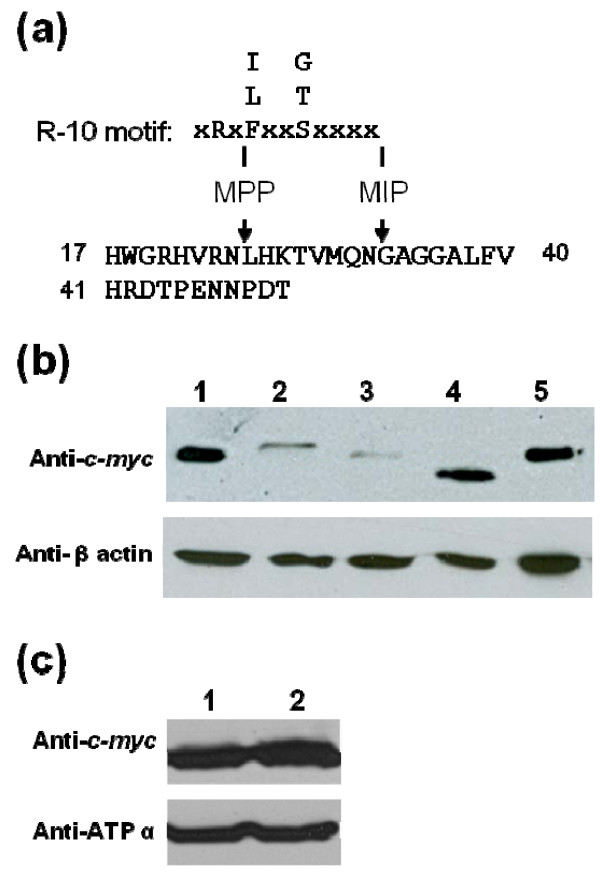
**Cleavage of the presequence occurs around residue 32 in the N-terminal region of NDUFV2**. (a) Two possible mitochondrial processing sites of NDUFV2 were predicted by the TargetP server [36]. The diagram shows a part of the N-terminal sequence of NDUFV2 (residues 17-51), with the MPP and MIP consensus cleavage sequence, R-10 motif (xRx↓(F/L/I)xx(S/T/G)xxxx↓), above it. The arrows indicate the expected MPP and MIP cleavage sites on NDUFV2. (b) The cleavage site of NDUFV2 *in vivo *is located around amino acid residue 32. Lanes 1-5, the total cell lysates of T-REx-293 transfected with the *c-myc*-tagged full-length NDUFV2 (lanes 1 and 5) and the *c-myc *-tagged NDUFV2 lacking the first 18, 32, and 50 residues respectively (lanes 2-4). Cell lysates were resolved by 15% SDS-PAGE, transferred, and probed with a mouse monoclonal anti-c-*myc *antibody. β-actin (42 kDa) was used as an internal control for Western blotting. (c) Mutation of the -10 arginine alone (i.e. R23A mutation) in the precursor has little effect on the formation of mature NDUFV2. Western blot analyses were conducted using mitochondrial extracts from T-REx-293 cells transiently transfected with the wild-type (lane 1) or NDUFV2 R23A mutant (lane 2) construct. The expressed proteins were detected by an anti-c-*myc *antibody. ATP synthase subunit α (ATP α) was used as a mitochondrial marker.

Most of the N-terminal presequences of mitochondrial matrix proteins are cleavable, primarily through the actions of MPP [28]. Typically, a single cleavage by MPP is sufficient for the maturation of most matrix protein precursors. However, when a not very well-defined octapeptide-containing precursor appears, two sequential cleavages carried out by MPP and MIP may occur (Figure 3a) (24). To determine whether NDUFV2 is processed by matrix proteases and estimate the approximate cleavage site of this protein *in vivo*, the full-length construct and three constructs encoding NDUFV2 suffering from an N-terminal truncation of a different length (△1-18 NDUFV2, △1-32 NDUFV2 and △1-50 NDUFV2) were transiently expressed in T-REx-293 cells and the sizes of these recombinant proteins were determined by Western blotting with the mouse anti-c-*myc *antibody (Figure 3b). The slower migration of the △1-18 NDUFV2 mutant protein than the wild-type NDUFV2 indicates that that the region containing the first 18 amino acids of NDUFV2 is essential for mitochondrial targeting of NDUFV2 and its subsequent proper processing. In contrast, the deletion mutant lacking the first 50 amino acids (△1-50 NDUFV2) was smaller than the natively processed NDUFV2, indicating that the native cleavage site must be in a position within the first 50 residues. Finally, the truncated NDUFV2 protein lacking the first 32 amino acids (△1-32 NDUFV2) had a similar migration rate with that of the natively processed NDUFV2. This finding strongly suggests that the final cleavage site for generation of the mature NDUFV2 protein is most likely located around residue 32 from the N-terminus of the precursor protein. It has to be specifically noted that the amount of both △1-18 and △1-32 NDUFV2 mutant proteins appears less when compared with that of the mature NDUFV2 or the △1-50 NDUFV2 mutant protein, indicating these two mutant proteins are less stable.

The cleavage site for MPP is usually indicated by an arginine residue at position -2 relative to the cleavage site, which is -10 relative to the amino terminus of the mature protein. To evaluate the involvement of this residue in NDUFV2 cleavage, we substituted the -10 arginine with alanine (i.e. R23A mutation) in the presequence of NDUFV2, and investigated the status of its processing in the mitochondrial fraction. As shown in Figure 3c, only one band with a similar intensity and size to that of the wild-type, mature NDUFV2 was present in the mitochondrial fraction. This result suggested that mutation of the -10 arginine alone in the precursor has little effect on the formation of mature NDUFV2.

### The first 22 amino acids in the N-terminal sequence of NDUFV2 were essential and efficient for mitochondrial targeting

**Figure 4:**
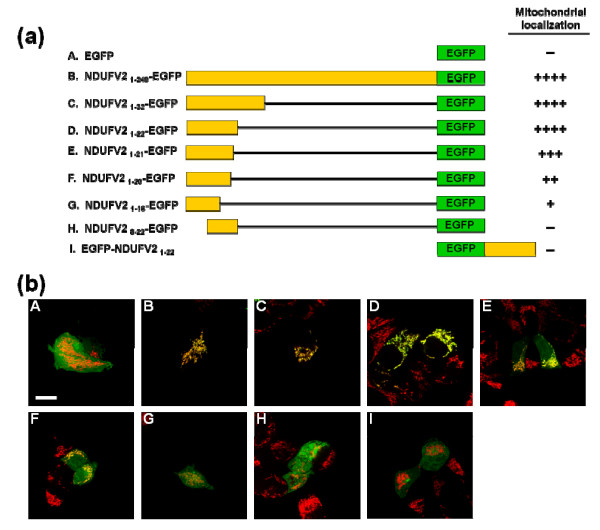
**The N-terminal 22-amino acid region of NDUFV2 is essential and efficient for mitochondrial targeting**. (a) The diagrammatic representation of EGFP fusion proteins carrying an NDUFV2 N-terminal peptide of a different length. A series of chimeric cDNA were constructed for expression of fusion proteins containing the full-length (NDUFV2_1-249 _-EGFP), N-terminal (NDUFV2_1-32 _-EGFP, NDUFV2_1-22 _-EGFP, NDUFV2_1-21 _-EGFP, NDUFV2_1-20 _-EGFP, NDUFV2_1-18 _-EGFP) or internal fragment (NDUFV2_8-22 _-EGFP) in the MTS of NDUFV2 with EGFP at the C-terminus or at the N-terminus (EGFP-NDUFV2_1-22_). The number of (+) symbols indicates the relative number of cells that exhibited EGFP fluorescence within the mitochondrial compartment in (b). The number of (+) symbols indicates that the proportion of cells exhibiting EGFP fluorescence have a typical punctuated staining pattern and mitochondrial colocalization in (b). The (++++) symbol indicates all of the EGFP fluorescence signals in transfected cells are fully colocalized with mitochondria. The (-) symbol indicates there is no cell producing EGFP fluorescence within the mitochondrial compartment. (b) The distribution of EGFP fusion proteins in transfected T-REx-293 cells was detected by EGFP fluorescence and mitochondria were labeled by Mito Tracker Red (red color). Only merged images are shown (colocalization of expressed protein and mitochondria is indicated by yellow signals). Photos A-I are corresponding to constructs A-I shown in (a). Scale bars = 10 * μ*m.

After identification of the MTS in NDUFV2 as well as the probable cleavage site of the protein, this study attempted to define the minimal region required for mitochondrial targeting. A series of chimeric constructs for expression of NDUFV2 MTS-EGFP fusion protein were generated (Figure 4a). As shown in Figure 4b, EGFP along without any targeting sequence addition was present throughout the cell, with some accumulation in the nucleus. On the other hand, EGFP fused with the full-length NDUFV2_1-249 _or the newly identified MTS (NDUFV2_1-32_) colocalized very well with that of the Mito Tracker Red dye, indicating that the first 32 amino acid acids in the N-terminus of NDUFV2 had a mitochondrial targeting ability comparable to that of the full-length NDUFV2. It was interesting to observe that the protein fragment containing the first 22 amino acid residues of NDUFV2 was sufficient to carry most (if not all) of the EGFP into mitochondria successfully, whereas the regions containing either the first 21 (NDUFV2_1-21 _-EGFP) or 20 (NDUFV2_1-20 _-EGFP) amino acid residues in the N-terminal sequence of NDUFV2 showed a much lower efficiency. When the first 18 amino acid residues were used as the signal peptide, the majority of mitochondrial targeting ability of this hybrid protein was lost (Figure 4b). The NDUFV2_8-22_-EGFP was also incapable of targeting to mitochondria. Together, these results indicate that the entire 1-22 residues are necessary for mitochondria targeting of NDUFV2.

Moreover, when the N-terminal 22 residues of NDUFV2 were moved to the C-terminus of EGFP, the mitochondrial targeting capability of this newly identified MTS functional region was completely lost (Figure 4b). This result implies that the MTS of NDUFV2 is directional and needs to be located at the N-terminus of NDUFV2 to be functional.

### Effects of basic residue and hydrophobic residue mutations in NDUFV2 MTS on mitochondrial targeting

**Figure 5:**
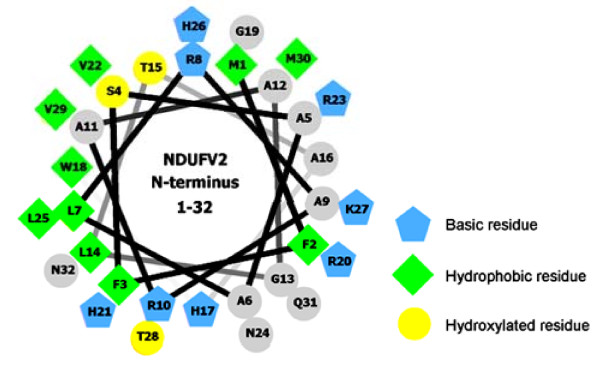
**The α-helical wheel diagram of the first 32 amino acids of NDUFV2**. The α-helical wheel model for the first 32 residues of NDUFV2 was constructed using Helical wheel projections [39]. The output presents the hydroxylated residues as yellow circles, hydrophobic residues as green diamonds, potentially basic (or positively charged) residues as blue pentagons, and the remaining residues as grey circles.

As shown in Figure 1a, the first 1-32 amino acids which we just demonstrated to function as the MTS have a net positive charge (contributing by 4 arginines, 1 lysine, 3 histidines, and the N-terminal methionine) but no acidic amino acids. Based on the Eisenberg method of hydrophobic moment calculation with Hmoment server [37], the MTS of NDUFV2 had a hydrophobic region roughly in the middle of the presequence. The secondary structure prediction using PSIPRED server [38] indicated that the first 1-32 residues of NDUFV2 contain two α-helical structures (one in residues 4-16, the other in residues 22-30) with one short coil structure in between (Figure 1b). When Helical Wheel Projections program [39] was applied to construct the α-helical wheel model for the N-terminus of NDUFV2, it was clear that the N-terminal region of NDUFV2 contains a typical amphiphilic structure with hydrophobic residues on one side and polar residues on the other side of the α-helix (Figure 5).

**Figure 6:**
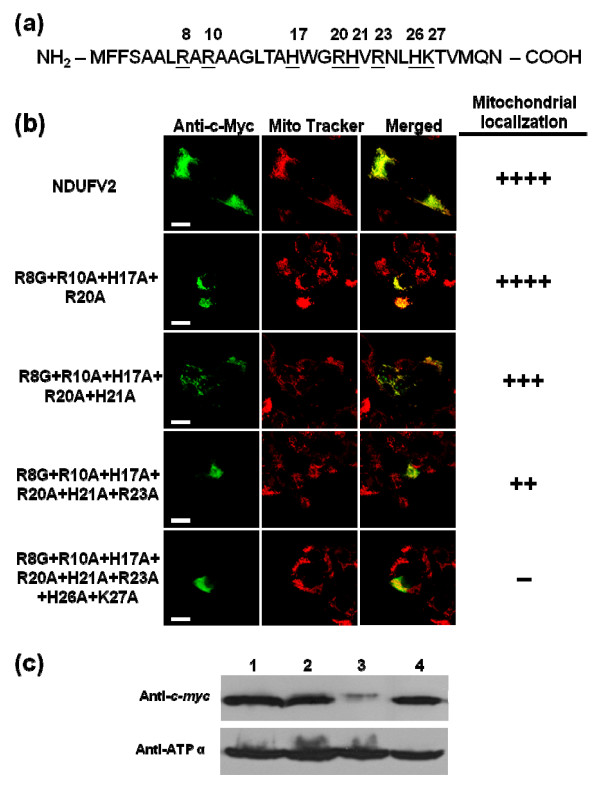
**Effects of basic residue mutation in NDUFV2 MTS on mitochondrial targeting**. (a) The sites of basic residue in NDUFV2 N-terminal 1-32 amino acids were underlined and marked. (b) The effect of basic residue mutation within the N-terminal region of NDUFV2 on mitochondrial targeting was evaluated by confocal image analyses. A series of point mutations targeting at arginine, lysine and histidine residues were introduced into NDUFV2 with the *c-myc *epitope tag and expressed in T-REx-293 cells. The expressed proteins with basic residue mutations were detected by an anti-c-*myc*-FITC antibody in transfected cells (green color), mitochondria were labeled by Mito Tracker Red (red color), and colocalization of expressed protein and mitochondria is shown as a merged image and indicated by yellow signals. The number of (+) symbols indicates that the proportion of cells exhibiting FITC fluorescence have a typical punctuated staining pattern and mitochondrial colocalization. The (++++) symbol indicates all of the FITC fluorescence signals in transfected cells are fully colocalized with mitochondria. The (-) symbol indicates that there is no cell producing FITC fluorescence within the mitochondrial compartment. Scale bars = 10 * μ*m. (c) The effect of basic residue mutation on mitochondrial targeting was investigated using subcellular fractionation and Western blotting analyses. Western blotting analyses were conducted using mitochondrial extracts from T-REx-293 cells transiently transfected with the wild-type (lane 1), quintuple mutant (R8G+R10A+H17A+R20A+H21A, lane 2), sextuple mutant (R8G+R10A+H17A+R20A+H21A+R23A, lane 3) or NDUFV2 R23A single-pointed mutant (R23A, lane 4) construct. The expressed proteins were detected by an anti-c-*myc *antibody. ATP synthase subunit α (ATP α) was used as a mitochondrial marker.

**Figure 7:**
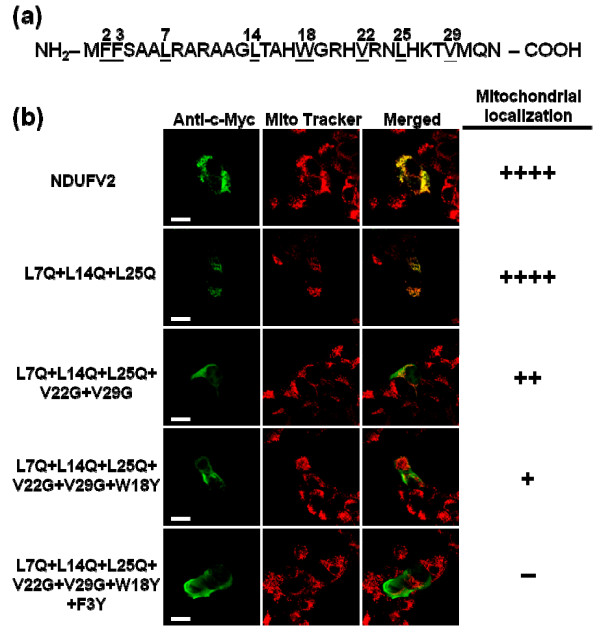
**Effects of hydrophobic residue mutation in NDUFV2 MTS on mitochondrial targeting**. (a) The sites of hydrophobic residue in NDUFV2 N-terminal 1-32 amino acids were underlined and marked. (b) The effect of hydrophobic residue mutation within the N-terminal region of NDUFV2 on mitochondrial targeting. A series of point mutations targeting at hydrophobic residues were introduced into NDUFV2 with the *c-myc *epitope tag and expressed in T-REx-293 cells. The expressed proteins with hydrophobic residue mutations were labeled by an anti-c-*myc*-FITC antibody in transfected cells (green color), mitochondria were labeled by Mito Tracker Red (red color), and colocalization of expressed protein and mitochondria is shown as a merged image and indicated by yellow signals. The number of (+) symbols indicates that the proportion of cells exhibiting FITC fluorescence have a typical punctuated staining pattern and mitochondrial colocalization. The (++++) symbol indicates all of the FITC fluorescence signals in transfected cells are fully colocalized with mitochondria. The (-) symbol indicates that there is no cell producing FITC fluorescence within the mitochondrial compartment. Scale bars = 10 * μ*m.

To examine the effect of basic, hydrophobic and hydroxylated residues within the N-terminal region of NDUFV2 on mitochondrial targeting, a site-directed mutagenesis methodology was applied systematically on these three groups of residues. The positively charged arginine, lysine and histidine residues were changed to non-charged residues, hydrophobic residues were replaced with hydrophilic residues and hydroxylated residues were substituted with residues without a hydroxyl group. The N-terminal 1-32 amino acids of NDUFV2 contain eight basic residues, including Arg8, Arg10, His17, Arg20, His21, Arg23, His26 and Lys27 (Figure 6a). Surprisingly, none of the substitutions at each individual basic amino acid residue affected the mitochondrial targeting function of the protein (data not shown). When three arginine residues (Arg8, Arg10 and Arg20) and one histidine (His17) were mutated at the same time to generate a quadruple mutant (Figure 6b), the resulting protein still yielded a mitochondrial localization pattern indistinguishable from that of the wild-type NDUFV2. However, when the fifth amino acid substitution (H21A) was introduced into the quadruple mutant, a slight reduction in the mitochondrial targeting was observed in the resulting protein (Figure 6b). With the introduction of increasing number of mutations in the basic residues, the resulting mutant gradually lost its capability of mitochondrial import. When all of the eight basic residues were mutated at the same time (the R8G+R10A+H17A+R20A+H21A+R23A+H26A+K27A octuple mutant), the ability of mitochondrial targeting of the protein was almost completely destroyed (Figure 6b). To further confirm the result obtained from confocal images, the strategy of subcellular fractionation, followed with quantitative analyses by Western blots was also applied on several mutants with a single-pointed mutation or multiple-pointed mutations on the basic residues. As shown in Figure 6c, the quantitative signals for the single-pointed mutant (R23A), quintuple mutant (R8G+R10A+H17A+R20A+H21A) and sextuple mutant (R8G+R10A+H17A+R20A+H21A+R23A) were 92%, 74% and 22%, respectively, of those of the wild-type T-REx-293 cell. This result is corresponding very well with the data derived from aforementioned confocal image analyses. Interestingly, when the same mutagenesis approach was applied to investigate the role of hydrophobic residues in the MTS of NDUFV2, a similar phenomenon was observed. Eight hydrophobic residues in total, including Phe2, Phe3, Leu7, Leu14, Trp18, Val22, Leu25 and Ala29 (Figure 7a), were selected for mutation to evaluate the effects of these changes on mitochondrial import but all of the single-point mutants showed an import efficiency comparable to that of the wild-type NDUFV2 (data not shown). A clear deficiency in mitochondrial targeting of these mutants was started to be observed when five hydrophobic residues in NDUFV2 N-terminus were mutated (the L7Q+L14Q+V22G+ L25Q +A29G quintuple mutant shown in Figure 7b). When 7 hydrophobic residues were mutated simultaneously (the L7Q+L14Q+V22G+ L25Q +A29G+W18Y+F3Y septuple mutant) the mitochondrial localization pattern was completely abolished. Finally, the only three hydroxylated residues, including Ser4, Thr15 and Thr28 in the NDUFV2 presequence were used for mutation, and the result showed that all of the mutations including single-, double- and triple-point mutations did not have a significant effect on the mitochondrial targeting of this protein (data not shown).

### Establishing the human disease mechanism of the early-onset hypertrophic cardiomyopathy and encephalopath

**Figure 8:**
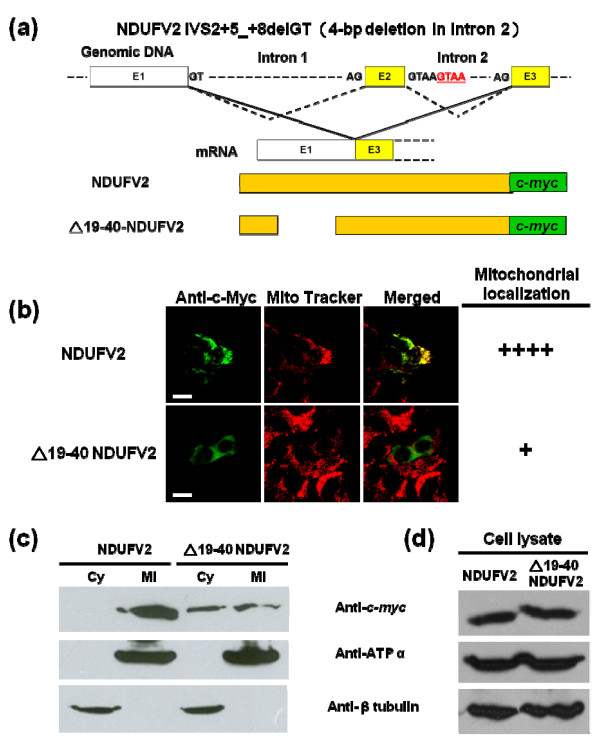
**The human pathogenic NDUFV2 deletion mutant lost most of its mitochondrial targeting ability**. (a) Schematic representation of the genomic structure of NDUFV2 in the first three exons. The 4-bp deletion of human pathogenic IVS2+5_+8delGTAA mutation is indicated by underlined red letters (GTAA). Dotted line represents the wild-type splicing form, and continuous line indicates the abnormal splicing form. Schematic structures of *c-myc *fusion proteins corresponding to the wild-type splicing form and the abnormal splicing form are also shown here. E1, E2 and E3 represent exon 1, exon 2 and exon 3, respectively. (b) The protein distribution patterns of wild-type and NDUFV2 IVS2+5_+8delGTAA mutant. The expressed proteins were labeled by an anti-c-*myc*-FITC antibody in transfected cells (green color), mitochondria were labeled by Mito Tracker Red (red color), and colocalization of expressed protein and mitochondria is shown as a merged image and indicated by yellow signals. The number of (+) symbols indicates that the proportion of cells exhibiting FITC fluorescence have a typical punctuated staining pattern and mitochondrial colocalization. The (++++) symbol indicates all of the FITC fluorescence signals in transfected cells are fully colocalized with mitochondria. Scale bars = 10 μm. (c) Subcellular localization of the wild-type and NDUFV2 pathogenic mutant. Western blot analyses were conducted using cytosolic (Cy) and mitochondrial (Mi) extracts from T-REx-293 cells transiently transfected with the wild-type or NDUFV2 pathogenic mutant construct. (c) The expression levels of the wild-type and the △19-40 NDUFV2 mutant proteins in the whole cell lysates. Western blot analyses were conducted using the whole cell lysates from T-REx-293 cells transiently transfected with the wild-type or the △19-40 NDUFV2 mutant construct. The expressed proteins were detected by an anti-c-*myc *antibody. β-tubulin was used as a cytosolic marker and ATP synthase subunit α (ATP α) was used as a mitochondrial marker.

The patients of early-onset hypertrophic cardiomyopathy and encephalopathy were shown to have a homozygous mutation, a 4-bp deletion in intron 2 (IVS2+5_+8delGTAA), in *NDUFV2 *gene [33]. This mutated gene finally produced a shortened NDUFV2 that lacks 19-40 residues due to a splicing donor site is affected (Figure 8a). The affected patients had a significant complex I deficiency and NDUFV2 missing. In a study using yeast *Y. lipolytica *as the model, the corresponding amino acids 17-32 from the orthologous NUHM protein have been deleted to mimic the disease condition. However, it was found that the resulting mutant produced a normal amount of NUHM, and this protein was fully assembled into complex I with a normal function [19]. This finding contradicted the situation described for the patients with early-onset hypertrophic cardiomyopathy and encephalopathy and thus prompted us to test the same mutation using the human cell model. The DNA fragment encoded residues 19-40 of NDUFV2 was removed from the wild-type NDUFV2 construct and the resulting plasmid was introduced into T-REx-293 cells for analysis. When confocal microscopy was used for tracking the expressed human disease associated NDUFV2 mutant protein (△19-40 NDUFV2), diffuse fluorescence was present throughout the cytoplasm and only a very limited mitochondrial localization was observed (Figure 8b). To confirm the immunofluorescent results, subcellular fractions prepared from T-Rex-293 cells transiently transfected with the wild-type and human disease mutant NDUFV2 constructs were applied for Western blotting analyses. As controls for proper cytosolic and mitochondrial separation, tubulin and ATP synthase α-subunit was used as a marker for the cytosol and mitochondria, respectively. In accordance with the immunofluorescent results, the wild-type NDUFV2 was found to be localized only in mitochondria whereas the △19-40 NDUFV2 mutant protein was detected mainly in the cytosol (Note: Equal amounts of total protein were loaded in each lane of gel and the △19-40 NDUFV2 mutant was expected to be less concentrated in the cytosol than in mitochondria) (Figure 8c). In addition, the size of △19-40 NDUFV2 (227 amino acids) observed in the Western blotting was slightly larger than that of the mature wild-type NDUFV2 (217 amino acids), implying that the △19-40 NDUFV2 mutant protein was not processed.

According to the original finding, fibroblasts from patients suffering from early-onset hypertrophic cardiomyopathy and encephalopathy had a significant reduction in the quality of NDUFV2 protein in mitochondria [33]. This observation agreed with the result of our aforementioned Western blotting analyses on the subcellular fractionation samples. However, it couldn't be completely ruled out that the reduced level of the mutant protein might also contribute to the pathophysiology of the disease. To evaluate this possibility, we conducted an experiment to investigate the expression levels of wild-type and mutant proteins in the whole cell lysates. As shown in Figure 8d, in spite of having a slightly larger size, the expression level of the △19-40 NDUFV2 mutant protein observed in the Western blotting was similar to that of the wild-type protein. This finding confirmed that the loss of mitochondrial import of the △19-40 NDUFV2 mutant protein is the major cause for early-onset hypertrophic cardiomyopathy and encephalopathy.

## Discussion

There are several lines of evidences indicating that applying an *in vitro *import system for mitochondrial targeting studies can lead to artificial results [40]. For this reason, *in vivo *analyses were used instead to investigate NDUFV2 import in this study. As the conventional subcellular fractionation requires large quantities of starting material which is very difficult to acquire using the transient transfection approach, confocal microscopy was applied as a convenient alternative to track the location of the transiently expressed protein. To confirm the immunofluorescence result, biochemical fractionation techniques was also adopted in the human pathogenic NDUFV2 deletion part of the study. The results derived from these two approaches were consistent with each other, indicating the confocal microscopy approach could be a reliable method to study the mitochondrial targeting of NDUFV2.

The N-terminal 1-32 amino acids of bovine 24-kDa and human NDUFV2 presequences have been suggested to contain the mitochondrial targeting sequence [31,32]. In this report, we experimentally characterized the human NDUFV2-MTS by deletion mapping and identified that the minimal sequence required for efficient mitochondrial targeting was located at the N-terminal amino acids 1-22. The location of this minimal MTS was directional: Addition of this sequence in the N-terminus of passenger protein EGFP promoted mitochondrial targeting of the fusion protein, but the phenomenon of mitochondrial localization was completely lost when it was appended to the C-terminus of EGFP. According to the result of secondary structure prediction, two α-helical structures (one in residues 4-16, the other in residues 22-30) connected by one short coil structure were evident in the signal peptide of the NDUFV2 (Figure 1b). The essentiality of the N-terminal amphiphilic α-helix in the MTS for mitochondrial targeting-recognition has been greatly discussed, and the importance of the α-helical structure in the C-terminal domain of MTS for mitochondrial processing-recognition has also been pointed out [41]. Based on the prediction, the N-terminal amino acids 1-22 contained a complete amphiphilic α-helix and our experimental results demonstrated that this N-terminal sequence was not only essential but also efficient for mitochondrial targeting of NDUFV2 and the passenger protein EGFP.

Most of the N-terminal presequences of mitochondrial matrix proteins are cleavable and removed to become mature proteins. When NDUFV2 was applied in MitoProt II [35] for MTS processing analyses, a cleavage site between residues 43 and 44 was predicted, whereas TargetP server [36] suggested a cleavage site between residues 32 and 33. The MitoProt II prediction fitted the R-2 motif rule, xRx↓x(S/x), with the presence of an arginine residue at the -2 position from the cleavage site (↓). As for the prediction from TargetP server, an R-10 motif, xRx↓(F/L/I)xx(S/T/G)xxxx↓[28], appeared in the presequence and implied that NDUFV2 could be cleaved first by MPP, followed by MIP with the arginine residue located at position -2 from the MPP cleavage site and -10 from the MIP cleavage site (Figure 3a). In this work we tested three deletions, △1-18, △1-32 and △1-50, in the N-terminal part of NDUFV2, and showed that NDUFV2 was processed *in vivo *probably by proteolytic removal of the N-terminal MTS at a cleavage site around amino acid residue 32 from the N-terminus of NDUFV2 precursor protein. Because the two cutting sites predicted for MPP and MIP are separated only by 8 amino acids and the Western blotting result of the natively processed NDUFV2 only showed a single band, it is very difficult for us to conclude whether this protein is processed through a single step or two-step cleavage. However, the result from our experiments fitted very well with the R-10 motif rule and implied that the precursor NDUFV2 might be sequentially processed by MPP and MIP in the mitochondrial matrix. Furthermore, our result also showed that mutation of the -10 arginine alone in the precursor has little effect on the formation of mature NDUFV2, indicating that NDUFV2 precursor without arginine at position -10 can still be the substrate for cleavage. This result is not surprising because it is well documented that site-directed mutagenesis of the -2, -3 or -10 arginine in different precursor molecules has displayed variable results, ranging from complete or partial inhibition of processing, generation of novel cleavage sites, to lack of any obvious effect [28]. In addition, it has been suggested that the structural elements in the presequence, or even in the mature portion of the protein may hide the most important determinants for mitochondrial processing of any given precursor [28].

It is well recognized that the positively charged residues in the MTS are important for mitochondrial targeting. However, in the present report, single-point mutations derived from eight basic residues in the NDUFV2 MTS had no marked effect on mitochondrial import, while their gradual mutation decreased the mitochondrial import efficacy of the protein. From this point of view, the targeting function of NDUFV2 MTS does not depend on a specific basic amino acid but may instead depend on the net positive charge and its overall presence. The NDUFV2 MTS, similar to most mitochondrial presequence, is predicted to maintain an N-terminal amphiphilic α-helical structure. The essentiality of the amphiphilicity of the N-terminal part of MTS is well recognized but the importance of the α-helix is controversial because some presequences do not have this structural property [30,42]. In addition, an experimental strategy which introduces point mutations to interrupt the predicted α-helical structure has a very high possibility to modify residues which are associated with the amphiphilic features of the MTS and thus makes the interpretation difficult. For this reason our present study was focused on the role of the hydrophilic and hydrophobic residues in NTUFV2 MTS. As shown in the prediction derived from the Helical Wheel Projections program [39], the N-terminus of NDUFV2 holds a typical amphiphilic structure with hydrophobic residues on one face and hydrophilic residues on the other face of the α-helix (Figure 5). The majority of these hydrophilic residues are actually those basic residues we just discussed that may contribute to the net positive charge of the NDUFV2 MTS. As for those hydrophobic residues, none of the single-point mutations had a significant effect on the import efficiency but the influence of these residues was gradually observed when the number of mutations was increased. All of these mutation results point to a conclusion that none of a single amino acid in the MTS of NDUFV2 is absolutely required for mitochondrial targeting of this protein, but maintaining a net positive charge and an amphiphilic structure with the overall balance and distribution of basic and hydrophobic amino acids is important for correct localization of NDUFV2.

Previous data from clinical researches indicated that the patients suffering from early-onset hypertrophic cardiomyopathy and encephalopathy disease frequently contain a 4-bp deletion in the *NDUFV2 *gene and produce a shortened NDUFV2 protein that lacks 19-40 residues [33]. When yeast *Y. lipolytica *was used as the model to simulate the deletion in the orthologous *NUHM *gene, it was found unexpectedly that the truncated protein lacking residues 17-32 residues was still fully assembled into complex I and carried out the normal function [19]. In contrast, our current results derived from the human cell model indicated that the majority of expressed human pathogenic NDUFV2 mutant with the disease corresponding deletion was unable to target to mitochondria. This deletion caused the NDUFV2 mutant to just have its first 18 residues of MTS remained in the N-terminus and thus significantly reduced its mitochondrial targeting ability. According to the prediction, this modification changed the secondary structure of NDUFV2 MTS greatly (Figure 1c). One half of the original first α-helix was remained but the second α-helix was completely lost in the MTS region of NDUFV2. Although a small amount of the NDUFV2 mutant could still be translocated into mitochondria, it could not be processed in the matrix due to the missing of its mitochondrial processing sites. This conclusion is also agreed with the mitochondrial targeting data from NDUFV2_1-18 _- EGFP constructs (Figure 4b). When functional NDUFV2 could not be imported to mitochondria by the defected MTS, complex I would lose its function in the energy transduction pathway. These data elucidate the deletion in the NDUFV2-MTS as a cause for early-onset hypertrophic cardiomyopathy and encephalopathy.

Nevertheless, why results derived from these two eukaryotic model systems have such big contradiction? According to the result of sequence identity and similarity analyzed by EMBOSS Pairwise Alignment Algorithms [43], though there is high conservation between human NDUFV2 and *Y. lipolytica *NUHM with 51.8% identity and 66.5% similarity, the identity and similarity for their presequences are only 18.8% and 28.1%, respectively. This comparison agrees with the general recognition that the MTS could be very diverse even between closely related orthologs. In addition, the functional MTS of NUHM has not been experimentally identified. We hypothesize that losing 17-32 amino acid residues in NUHM does not disrupt the functional MTS region of this protein so that this mutant protein could still be transported to mitochondria and retain its normal assembly and activities in complex I. In order to support this hypothesis, we used the MitoProt server [35] to predict the possible MTS in NUHM protein and the result suggested that it is located at the first 1-16 amino acids of NUHM and carries a R-3 cleavage motif (xRx(Y/x)↓(S/A/x)x). In addition, we also applied the Predotar server [44] to predict the location of MTS in the mutant NUHM, and found that the △17-32 NUHM mutant still has a high degree of mitochondrial targeting score similar to that of the intact NUHM (Table 1). Also, it was reported that human *NDUFV2 *cDNA could not complement a NUHM subunit deletion in the *Y. lipolytica *model study. Moreover, the N-terminal region of △17-32 NUHM mutant did not lose its amphiphilic α-helical pattern predicted by Helical Wheel Projections program (data not shown) [39]. The real situation of mitochondrial targeting in *Y. lipolytica *requires further experiments to confirm.

**Table 1:** Prediction of subcellular location of NDUFV2-related proteins by the Predotar v.1.03 server [44]

	Mitochondria	Endoplasmic reticulum	Elsewhere
	
Protein	Possibility		
NUHM	0.95	0.01	0.05
△17-32 NUHM	0.94	0.01	0.06
NDUFV2	0.85	0.01	0.15
△19-40 NDUFV2	0.46	0.01	0.54
△1-22 NDUFV2	0.04	0.01	0.95

Pathogenic mutations found to affect protein localization are called mislocalization mutations. Modification in mitochondrial targeting signals can cause a protein not arriving at its final destination and eventually lead to human diseases. An arginine-to-proline substitution in the MTS of E1α subunit of the mitochondrial matrix protein complex pyruvate dehydrogenase (PDH) was the first reported case related to the malfunction of a mitochondrial targeting signal [45]. Infants and children carrying this mutation showed a significant X-linked PDH deficiency and developed primary lactic acidosis which led to severe microcephaly and cerebral atrophy. Biochemical analyses indicated the PDH activity and the level of PDH E1α protein were dramatically reduced in cultured skin fibroblasts. A similar but in the opposite twist of disorders associated with mitochondrial targeting is that some diseases are caused by the mislocalization of a protein which is normally not present in mitochondria. One example is the association of mistargeting of the peroxisomal alanine:glyoxylate aminotransferase (AGT) to mitochondria with patients having primary hyperoxaluria type 1. A single mutation (proline-to-leucine) in the AGT activates a cryptic MTS, that accompanying with the second mutation (glycine-to-arginine) in the other part of the protein, changes the AGT targeting from peroxisomes to mitochondria [46]. This mislocalization of AGT disrupts peroxisomal function and finally leads to diseases. These examples support our argument that the mislocalization of NDUFV2 caused by the IVS2+5_+8delGTAA mutation in *NDUFV2 *gene is associated with early-onset hypertrophic cardiomyopathy and encephalopathy.

## Conclusions

In conclusion, the MTS of NDUFV2 is located at the N-terminus of the precursor protein and is proteolytically removed at a cleavage site around amino acid residue 32. The first 22 residues of NDUFV2 are essential and efficient to carry the passenger protein into mitochondria and the location of this minimal MTS is directional. None of a single amino acid in the MTS of NDUFV2 is absolutely required for mitochondrial targeting of this protein, but maintaining a net positive charge and an amphiphilic structure with the overall balance and distribution of basic and hydrophobic amino acids are important. The results of human disease cell model establish that the impairment of mitochondrial localization of NDUFV2 as a mechanistic basis for early-onset hypertrophic cardiomyopathy and encephalopathy.

## Abbreviations

DAPI: diamidino-2-phenylindole; DMEM: Dulbecco's modified Eagle's medium; EGFP: enhanced green fluorescence protein; EPR: electron paramagnetic resonance; ETC: electron transport chain; FBS: fetal bovine serum; FITC: fluorescein isothiocyanate; FMN: flavin mononucleotide; MIP: mitochondrial intermediate peptidase; MMP: mitochondrial membrane potential; MPP: mitochondrial processing peptidase; MTS: mitochondrial targeting sequence; NDUFV2: NADH dehydrogenase (ubiquinone) flavoprotein 2; OXPHOS: oxidative phosphorylation system; TIM: translocase of inner membrane; TOM: translocase of outer membrane.

## Competing interests

The authors declare that they have no competing interests.

## Authors' contributions

HYL contributed to the study design, did most of the experiments, and wrote the first draft of the manuscript. PCL and KTC participated in the design and conducted subcellular fractionation experiments and Western blotting analyses. MCK, the correspondence author, organized the whole study design, team discussion, and final revision of this paper. All authors read and approved the final manuscript.

## Supplementary Material

Additional file 1 **Sequences of the primers used in this study**. Click here for file
