# Supplementary material for: Mitochondrial targeting of human NADH dehydrogenase (ubiquinone) flavoprotein 2 (NDUFV2) and its association with early-onset hypertrophic cardiomyopathy and encephalopathy
Source: J Biomed Sci. 2011 May 6;18(1):29. doi: 10.1186/1423-0127-18-29 (PMC3117770; doi:10.1186/1423-0127-18-29)
Supplement: Additional file 1 — Sequences of the primers used in this study. [file 1423-0127-18-29-S1.PDF]

Additional file 1

Sequences of the primers used in this study:

(1) Primers used for construction of plasmids expressing the full-length NDUFV2 protein.

Primer name	Sequence (5' → 3')
NDUFV2- F	TAGAATTCCCCGCCATGTTCTTCTCCG
NDUFV2- R	TACTCGA GAAGGCCTGCTTGTACACCAAATC

(2) Primers used for construction of plasmids expressing truncated NDUFV2 proteins.

**Construct**	Primer name	Sequence (5' → 3')
**delNDUFV2 1-32**	dMTS NDUFV2-F1	CGAATTCATGGGAGCTGGAGGAGCTTTA
**delNDUFV2 1-20**	dMTS NDUFV2-F2	CGAATTCATGCATGTAAGGAATTTGCAT
**delNDUFV2 1-18**	dMTS NDUFV2-F3	CGAATTCATGGGAAGACATGTAAGGAAT
**delNDUFV2 1-40**	dMTS NDUFV2-F4	CGAATTCATGCACAGAGATACTCCTGAG
**delNDUFV2 1-50**	dMTS NDUFV2-F5	CGAATTCATGACTCCATTTGATTTCACAC
**Reverse primer**	dMTS NDUFV2-R1	GCTCGAGAAGGCCTGCTTGTACACC
**del183-249 NDUFV2**	NDUFV2-*EcoR*1-F	CGAATTCCCCGCCATGTTCTTCTCCGC
	NDUFV2-Cys motif-*Xho*I-R	ACTCGAGGTTCACACAGGCCCCTAAAC
**del198-249 NDUFV2**	NDUFV2-*EcoR*I-F	CGAATTCCCCGCCATGTTCTTCTCCGC
	NDUFV2-Cys motif-*Xho*I-R2	GCTCGAGTGTCAAATCCTCATAGTAATTG

(3) Primers used for construction of plasmids expressing NDUFV2 mutants with the human pathogenic IVS2+5_+8delGTAA mutation.

Primer name	Sequence (5' → 3')
dE2-Not1-R	GCGGCCGCACCAGTGGGCGGTGAGGCC
dE2-Not1-F	GCGGCCGCCACAGAGATACTCCTGAG
ddE2-F	GCTGGCCTCACCGCCCACTGGCACAGAGATACTCCTGAGAATAAC
ddE2-R	GTTATTCTCAGGAGTATCTCTGTGCCAGTGGGCGGTGAGGCCAGC
NDUFV2-ddE2-m-F	GGGCCAAGGAGTGGACGCTTCTCTTGTG
NDUFV2-ddE2-m-R	CACAAGAGAAGCGTCCACTCCTTGGCCC

(4) Primers used for construction of plasmids expressing various lengths of NDUFV2-EGFP fusion proteins.

**Construct**	Primer name	Sequence (5' → 3')
	NDUFV2-MTS- N3-1F	CCTCGAGATGTTCTTCTCCGCGGCG
	NDUFV2-MTS-N3-2F	CCTCGAGATGCGGGCCCGGGCGGCTGGC
**NDUFV2-1~18 (1F1R)**	NDUFV2-MTS- N3-1R	CGAATTCGCCCAGTGGGCGGTGAGGCC
**NDUFV2-1~40 (1F2R)**	NDUFV2-MTS- N3-2R	CGAATTCGCCACAAATAAAGCTCCTCC
**NDUFV2-1~32 (1F3R)**	NDUFV2-MTS- N3-3R	CGAATTCGCATTTTGCATAGCTGTCTT
**NDUFV2-1~249 (1F4R)**	NDUFV2-MTS- N3-4R	CGAATTCGCAAGGCCTGCTTGTACACC
**NDUFV2-1~27 (1F5R)**	NDUFV2-MTS- N3-5R	CGAATTCGCCTTATGCAAATTCCTTAC
**NDUFV2-1~23 (1F6R)**	NDUFV2-MTS- N3-6R	CGAATTCGCCCTTACATGTCTTCCCCAG
**NDUFV2-1~22 (1F8R)**	NDUFV2-MTS- N3-8R	CGAATTCGCTACATGTCTTCCCCAGTG
**NDUFV2-1~20 (1F9R)**	NDUFV2-MTS- N3-9R	CGAATTCGCTCTTCCCCAGTGGGCGGTG
**NDUFV2-1~21 (1F10R)**	NDUFV2-MTS- N3-10R	CGAATTCGCATGTCTTCCCCAGTGGGC
**NDUFV2-8~22 (2F8R)**	NDUFV2-MTS- N3-8R	CGAATTCGCTACATGTCTTCCCCAGTG

(5) Primers used for construction of plasmids expressing NDUFV2 mutant with hydroxylated residue mutations.

Construct	Primer name	Sequence (5' → 3')
**T15A**	NDUFV2-MTS T15A-F	GGCGGCTGGCCTCGCAGCCCACTGGGGAAG
	NDUFV2-MTS T15A-R	CTTCCCCAGTGGGCTGCGAGGCCAGCCGCC
**S4G**	NDUFV2-MTS-S4G-F	CCGCCATGTTCTTCGGAGCGGCGCTCCGGG
	NDUFV2-MTS-S4G-R	CCCGGAGCGCCGCTCCGAAGAACATGGCGG
**T28A**	NDUFV2-MTS-T28A-F	GGAATTTGCATAAGGCAGTTATGCAAAATG
	NDUFV2-MTS-T28A-R	CATTTTGCATAACTGCCTTATGCAAATTCC

(6) Primers used for construction of plasmids expressing NDUFV2 mutants with basic residue mutations.

Construct	Primer name	Sequence (5' → 3')
**R8G**	NDUFV2-MTS CCG21,22,24AGT(R8G)-F	TCTCCGCGGCGCTAGGTGCCCGGGCGGCTG
	NDUFV2-MTS CCG21,22,24AGT-(R8G)R	CAGCCGCCCGGGCACCTAGCGCCGCGGAGA
R10A+H17A+**R8G**	NDUFV2-MTS-R8G-2F	TCTCCGCGGCGCTAGGTGCCGCGGCGGCTG
	NDUFV2-MTS-R8G-2R	CAGCCGCCGCGGCACCTAGCGCCGCGGAGA
**R10A**	NDUFV2-MTS CG28,29GC(R10A)-F	CGGCGCTCCGGGCCGCGGCGGCTGGCCTCA
	NDUFV2-MTS CG28,29GC(R10A)-R	TGAGGCCAGCCGCCGCGGCCCGGAGCGCCG
R8G+H17A+**R10A**	NDUFV2-MTS-R10A-F2	CGGCGCTAGGTGCCGCGGCGGCTGGCCTCA
	NDUFV2-MTS-R10A-R2	TGAGGCCAGCCGCCGCGGCACCTAGCGCCG
**H17A**	NDUFV2-MTS CG49,50GC(H17A)-F	CTGGCCTCACCGCCGCCTGGGGAAGACATG
	NDUFV2-MTS CG49,50GC(H17A)-R	CATGTCTTCCCCAGGCGGCGGTGAGGCCAG

R8G+R10A+H17A+**H21A**	NDUFV2-MTS-H21A-F	CCGCCTGGGGAAGAGCTGTAAGGAATTTGC
	NDUFV2-MTS-H21A-R	GCAAATTCCTTACAGCTCTTCCCCAGGCGG
R8G+R10A+H17A+H21A+**R20A**	NDUFV2-MTS-R20A-F	CGCCGCCTGGGGAGCAGCTGTAAGGAATT
	NDUFV2-MTS-R20A-R	AATTCCTTACAGCTGCTCCCCAGGCGGCG
R8G+R10A+H17A+H21A+R20A+**R23A**	NDUFV2-MTS-R23A-F	GGGAGCAGCTGTAGCGAATTTGCATAAG
	NDUFV2-MTS-R23A-R	CTTATGCAAATTCGCTACAGCTGCTCCC
R8G+R10A+H17A+H21A+R20A+R23A+**H26A**	NDUFV2-H26A-F	CTGTAGCGAATTTGGCTAAGACAGTTATG
	NDUFV2-H26A-R	CATAACTGTCTTAGCCAAATTCGCTACAG
R8G+R10A+H17A+H21A+R20A+R23A+H26A+**K27A**	NDUFV2-K27A-F	GTAGCGAATTTGGCTGCGACAGTTATGCAA
	NDUFV2-K27A-R	TTGCATAACTGTCGCAGCCAAATTCGCTAC

(7) Primers used for construction of plasmids expressing NDUFV2 mutants with hydrophobic residue mutations.

**Construct**	Primer name	Sequence (5’ → 3’)
L7Q	NDUFV2-MTS-L7Q-F	CTTCTCCGCGGCGCAACGGGCCCGGGCGGC
	NDUFV2-MTS-L7Q-R	GCCGCCCGGGCCCGTTGCGCCGCGGAGAAG
L14Q	NDUFV2-MTS-L14Q-F	CCGGGCGGCTGGCCAAACCGCCCACTGGG
	NDUFV2-MTS-L14Q-R	CCCAGTGGGCGGTTTGGCCAGCCGCCCGG
L25Q	NDUFV2-MTS-L25Q-F	GACATGTAAGGAATCAGCATAAGACAGTTA
	NDUFV2-MTS-L25Q-R	TAACTGTCTTATGCTGATTCCTTACATGTC
V22G	NDUFV2-V22G-F	CTGGGGAAGACATGGAAGGAATCAGCATA
	NDUFV2-V22G-R	TATGCTGATTCCTTCCATGTCTTCCCCAG
V29G	NDUFV2-V29G-F	CAGCATAAGACAGGTATGCAAAATGGAG

	NDUFV2-V29G-R	CTCCATTTTGCATACCTGTCTTATGCTG
L7,14,25Q+**V22G**	NDUFV2-V22G-F2	CTGGGGAAGACATGGAAGGAATTTGCATA
	NDUFV2-V22G-R2	TATGCAAATTCCTTCCATGTCTTCCCCAG
L7,14,25Q+V22,**29**G	NDUFV2-V29G-F2	TTGCATAAGACAGGTATGCAAAATGGAG
	NDUFV2-V29G-R2	CTCCATTTTGCATACCTGTCTTATGCAA
F2Y	NDUFV2-F2Y-F	GAATTCCCCGCCATGTACTTCTCCGCGGCG
	NDUFV2-F2Y-R	CGCCGCGGAGAAGTACATGGCGGGGAATTC
F3Y	NDUFV2-F3Y-F2	CCCCGCCATGTTCTACTCCGCGGCGCTCCG
	NDUFV2-F3Y-R2	CGGAGCGCCGCGGAGTAGAACATGGCGGGG
W18Y	NDUFV2-W18Y-F2	CTCACCGCCCACTATGGAAGACATGTAAG
	NDUFV2-W18Y-R2	CTTACATGTCTTCCATAGTGGGCGGTGAG
L7,14,25Q+V22,29G+**W18Y**	NDUFV2-W18Y-F	CAAACCGCCCACTATGGAAGACATGGAAG
	NDUFV2-W18Y-R	CTTCCATGTCTTCCATAGTGGGCGGTTTG
L7,14,25Q+V22,29G+W18Y+**F3Y**	NDUFV2-F3Y-F	CCCCGCCATGTTCTACTCCGCGGCGCAAC
	NDUFV2-F3Y-R	GTTGCGCCGCGGAGTAGAACATGGCGGGG
